# Neoadjuvant immunotherapy for NSCLC: superior combination strategies, optimal treatment cycles, and predictive indicators from a Bayesian meta-analysis

**DOI:** 10.3389/fimmu.2025.1548665

**Published:** 2025-03-27

**Authors:** Yubingxue Liu, Jianlin Long, Huan Deng, Wen Chen

**Affiliations:** ^1^ Department of Health Examination and Oncology Screening Center, Chongqing University Cancer Hospital and Chongqing Cancer Institute, Chongqing, China; ^2^ Department of Medical Oncology, Chongqing University Cancer Hospital and Chongqing Cancer Institute, Chongqing, China

**Keywords:** neoadjuvant immunotherapy, non-small cell lung cancer, treatment cycles, surrogate endpoints, meta-analysis

## Abstract

**Background:**

Neoadjuvant immune checkpoint inhibitors (ICIs) have emerged as a promising treatment strategy for resectable non-small cell lung cancer (NSCLC). However, optimal combination strategies, treatment cycles, and predictive indicators for long-term outcomes remain unclear. This study aimed to evaluate the efficacy of various neoadjuvant ICI-based therapies in resectable NSCLC, identify the optimal treatment cycles for neoadjuvant immunochemotherapy, and assess the prognostic value of pathological complete response (pCR) and major pathological response (MPR) for event-free survival (EFS).

**Methods:**

A systematic literature search was conducted in PubMed, EMBASE, Cochrane CENTRAL, and Web of Science, including studies published up to October 2024. Bayesian models were used to analyze the efficacy of different ICI-based treatment combinations, assess the impact of immunochemotherapy cycles on MPR and pCR, and examine the predictive value of MPR and pCR for EFS.

**Results:**

Data from 34 studies were included, consisting of 32 single-arm studies (reported in 26 papers) and 8 RCTs, involving 4,593 patients. Immunochemotherapy combined with anti-angiogenesis agents was the most effective treatment strategy, significantly improving both MPR and pCR. No significant improvement in efficacy was observed when the number of neoadjuvant immunochemotherapy cycles exceeded 3 cycles. Both MPR and pCR were strong predictors of EFS. MPR showed a stronger negative correlation with event risk compared to pCR, with a log (HR) of -2.110 (95% CI: -4.150, -0.071) for MPR, and a log (HR) of -1.665 (95% CI: -2.419, -0.992) for pCR.

**Conclusion:**

Neoadjuvant immunochemotherapy combined with anti-angiogenesis agents appears to be a highly effective strategy for resectable NSCLC. Three cycles of neoadjuvant immunochemotherapy demonstrated optimal efficacy in this study. Both MPR and pCR are valuable prognostic indicators for EFS, with MPR showing a stronger predictive value. These findings offer important insights for optimizing treatment strategies and informing clinical decision-making in resectable NSCLC.

**Systematic review registration:**

PROSPERO, identifier CRD42024592346.

## Introduction

Lung cancer remains the leading cause of cancer-related mortality worldwide, with non-small cell lung cancer (NSCLC) accounting for approximately 85% of cases (1). While surgical resection is the cornerstone treatment for early-stage NSCLC, only about 25% of patients are diagnosed with resectable disease (2). Moreover, even after surgery, recurrence or metastasis occurs in 30% to 55% of cases (3). This underscores the critical need for effective preoperative strategies, particularly for locally advanced NSCLC, to improve resectability and address micrometastatic disease.

Neoadjuvant immune checkpoint inhibitors (ICIs), often combined with chemotherapy, have shown promise in reshaping the treatment landscape for resectable NSCLC. Landmark phase III trials such as CheckMate 816 (4), KEYNOTE-671 (5), and Neotorch (6) have demonstrated significant improvements in key outcomes, including pathological complete response (pCR), major pathological response (MPR), and event-free survival (EFS), establishing neoadjuvant chemoimmunotherapy as a transformative strategy.

Despite these advances, important questions remain unanswered regarding the optimization of ICI-based neoadjuvant therapies. The relative effectiveness of different ICI-based combinations, such as ICIs with chemoradiotherapy or anti-angiogenesis agents, is still unclear. Similarly, the ideal number of treatment cycles has yet to be determined. Furthermore, while MPR and pCR are commonly used to evaluate neoadjuvant therapy responses, their predictive value for long-term outcomes such as EFS warrants further exploration.

Existing meta-analyses are limited by heterogeneity in study designs, populations, and protocols, often introducing biases. Additionally, most fail to integrate data from single-arm studies and randomized controlled trials (RCTs), hindering comprehensive comparisons of neoadjuvant strategies. To overcome these challenges, this study employs a Bayesian hierarchical meta-analysis, a statistical approach well-suited for synthesizing data from heterogeneous sources, such as single-arm studies and RCTs. Bayesian framework incorporates prior knowledge and provides probabilistic estimates, offering greater flexibility in handling complex data structures and uncertainty (7). By leveraging this approach, we systematically evaluate the comparative efficacy of various ICI-based neoadjuvant strategies, optimize treatment cycles, and investigate the prognostic value of MPR and pCR for EFS. This study aims to provide robust evidence to guide clinical decision-making and improve outcomes for patients with resectable NSCLC.

## Methods

### Study design

This study adhered to the Preferred Reporting Items for Systematic Reviews and Meta-Analyses (PRISMA) guidelines and its extension for network meta-analyses (PRISMA-NMA). The protocol was registered in PROSPERO (registration number: CRD42024592346). The primary objective was to compare the efficacy of different neoadjuvant ICI-based therapies in resectable NSCLC, focusing on pCR and MPR. Secondary objectives included evaluating the impact of different neoadjuvant therapy cycles of ICIs plus chemotherapy on MPR and pCR, as well as assessing the predictive value of MPR and pCR for EFS.

### Search strategy and data collection

A systematic literature search was conducted in PubMed, EMBASE, Cochrane CENTRAL, and Web of Science for studies published up to October 30, 2024. Search terms combined the following keywords: “neoadjuvant,” “immune checkpoint inhibitors,” “chemotherapy,” “non-small cell lung cancer,” and “clinical trial.” Reference lists of included studies were manually reviewed for additional relevant studies. Two independent reviewers screened the titles and abstracts, and duplicates were removed using EndNote. Full texts of potentially eligible studies were retrieved for further assessment. Discrepancies between reviewers were resolved through discussion with a third reviewer.

### Inclusion and exclusion criteria

Studies were included if they involved patients with histologically confirmed resectable NSCLC (stages IA-IIIB) without prior systemic therapy, and interventions included neoadjuvant ICIs alone or with chemotherapy, other ICIs, radiotherapy, chemoradiotherapy, or anti-angiogenesis agents. For RCTs, the control group was neoadjuvant chemotherapy or placebo. Eligible studies reported pCR and/or MPR and were phase II single-arm studies or phase II/III RCTs.

Studies were excluded if they were retrospective, case reports, reviews, meta-analyses, or contained duplicated data. In the case of duplicated data, preference was given to the most comprehensive or recent study with larger sample sizes, longer follow-up, and more complete data. Studies not reporting pCR or MPR outcomes or lacking standardized definitions (e.g., MPR as <10% residual viable tumor) were also excluded.

### Data extraction

Study quality was assessed using standardized tools appropriate for the study design (RoB 2.0 for RCTs and NOS for single-arm studies). Two independent reviewers then extracted data, including study design (RCTs or single-arm), registration number, sample size, race/region, tumor stage, treatment arms, therapy cycles, and outcomes such as MPR (%), pCR (%), and 24-month EFS rates. Hazard ratios (HRs) comparing EFS between MPR vs. non-MPR and pCR vs. non-pCR were also collected. Any discrepancies were resolved through discussion with a third reviewer.

### Statistical analysis

Various statistical methods were used to evaluate the efficacy of neoadjuvant immunotherapy strategies, with Bayesian hierarchical models applied to account for differences between single-arm studies and RCTs.

In single-arm studies, treatment effects were estimated flexibly using prior distributions, where each group’s effect followed a normal distribution centered around a global baseline effect (μ_global ∼ N (-0.85, 0.1)), with a shrinkage estimate (μ[j] ∼ N (μ_global, τ)) to control for between-group variability, and τ modeled using a Gamma prior (τ ∼ Gamma (0.01, 0.01)). In the joint analysis of single-arm studies and RCTs, differences between study types were explicitly accounted for, with each treatment effect (α_j) following a normal distribution (N (0, n_j)) where variance was determined by sample size—larger RCTs had higher precision while smaller single-arm studies had greater variance. Additionally, study-type effects (β_k) followed a wide normal prior (β_k ∼ N (0, 100)), allowing data-driven estimation of treatment effect differences between study types. This modeling approach ensures a balanced assessment of treatment efficacy while maintaining statistical rigor.

Bayesian hierarchical models with Beta regression (logit link) were used to evaluate the impact of race on MPR and pCR, with race categorized as White Dominant (White ≥ 50%), Asian Dominant (Asian ≥ 50%), and Mixed (no single race ≥ 50%). Race was treated as a fixed effect, and study as a random effect. Sample size was log-transformed and used as a weight. The models estimated treatment effects with posterior distributions to assess potential racial and drug-related differences.

To analyze the impact of different neoadjuvant chemoimmunotherapy cycles on MPR and pCR. Patients were categorized into three groups based on the number of treatment cycles received: three cycles (baseline group), mixed cycles (comprising 2–3, 2–4, and 3–4 cycle combinations), and four cycles. Using the three-cycle group as the reference, we estimated the log odds ratio (LogOR) and its 95% confidence interval for MPR and pCR, where a negative LogOR indicated reduced treatment efficacy. The probability distributions of MPR and pCR were visualized using probability bar plots.

To examine the surrogacy potential of MPR and pCR for EFS, weighted linear regression was performed, with bootstrap methods estimating confidence intervals for regression parameters. Analyses were conducted in R (version 4.4.1) using JAGS, employing Markov Chain Monte Carlo methods to sample posterior distributions. Convergence diagnostics, including Gelman-Rubin statistics values all equal to 1.00, indicating complete convergence and trace plots, confirmed model robustness and reliable sampling. Key results were visualized through forest plots, heatmaps, and scatterplots.

### Publication bias

This study used a Bayesian model to generate posterior effect size distributions and employed the Wilcoxon rank-sum test to compare single-arm studies and RCTs. Density plots were created to visualize differences and overlaps, assessing potential publication bias.

## Results

### Study selection

**Figure 1:**
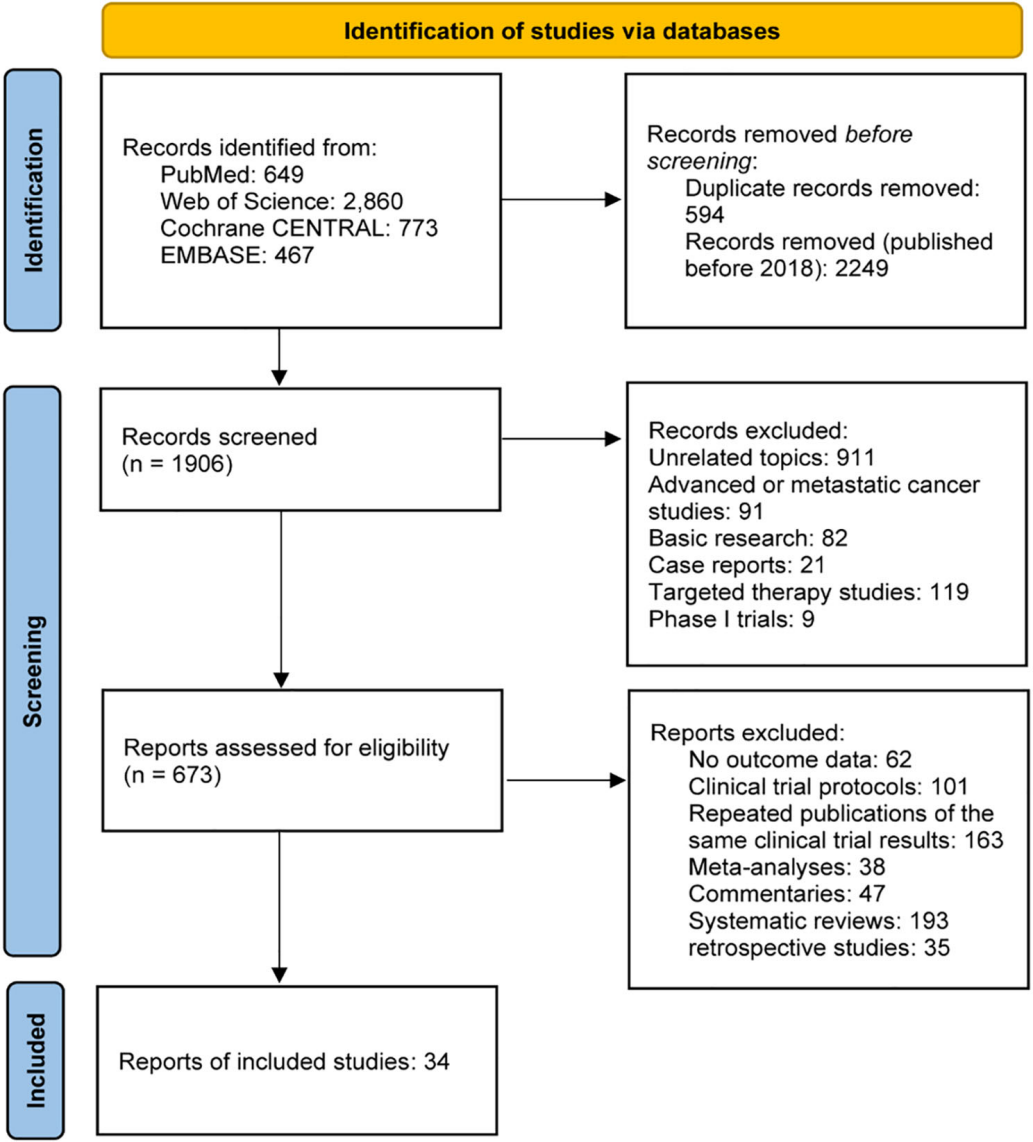
PRISMA Flow diagram of study selection process.

The initial search identified 4,749 records from PubMed (649), Web of Science (2,860), Cochrane CENTRAL (773), and EMBASE (467). After excluding 2,249 studies published before 2018 and 594 duplicates, 1,906 unique records remained. Further screening removed phase I trials (9), targeted therapy studies (119), case reports (21), retrospective studies (35), meta-analyses (38), basic research (82), trial protocols (101), commentaries (47), duplicate trial publications (163), studies on advanced/metastatic cancer (91), unrelated topics (911), and those lacking outcome data (62). In total, 34 studies (4,593 patients) were included in the meta-analysis: 32 single-arm studies (from 26 papers) and 8 RCTs, as shown in **Figure 1**. Study details are in **Tables 1** and **2**.

**Table 1:** Summary of single-arm studies evaluating neoadjuvant immunotherapy and combination regimens in NSCLC.

Type of treatment	Registration Number	Author and Year	Study Phase	Race	Sample Size	Patient Stage	Treatment	Therapy Cycles	MPR (%)	pCR (%)	24m EFS (%)
Immunotherapy Alone	NCT02259621	Forde PM, et al. 2018 (21).	II	White	21	I-IIIA	Nivolumab	2	45	15	–
	NCT03158129	Cascone T, et al. 2023 (22).	II	White (91%)/Black (4%)/Asian (5%)	23	I-IIIA	Nivolumab	3	21.7	8.7	–
	NCT03794544	Cascone T, et al. 2023. (23)	II	White	27	IA3-IIIA	Durvalumab	1	12.5	4.2	–
	NCT03030131	Wislez M, et al. 2022. (24)	II	White	46	IB-IIIA(Non-N2)	Durvalumab	3	18.6	7	–
	NCT02904954	Altorki NK, et al. 2021 (25).	II	White (81%)/Black(11%)/Asian(8%)	30	I-IIIA	Durvalumab	2	6.7	0	69 (51.4-87)
	NCT02994576	Besse B, et al. 2020 (26).	II	White	30	IA3-IIIA	Atezolizumab	1	13.3	13	–
	NCT02927301	Chaft JE, et al. 2022 (27).	II	White (81%)/Black (7%)/Asian (5%)/Unknown (7%)	181	IB-IIIB	Atezolizumab	2	20.8	6.9	76(68.85-83.15)
	NCT02818920	Tong BC, et al. 2022 (28).	II	White (97%)/Black (3%)	30	IB-IIIA	Pembrolizumab	2	28	12	–
Immunotherapy + Radiotherapy	NCT02904954	Altorki NK, et al. 2021 (25).	II	White (75%)/Black (13%)/Asian(11%)	30	I-IIIA	Stereotactic body radiotherapy + Durvalumab	2	53.3	26.7	85 (70.7-98.5)
Immunotherapy + Chemoradiotherapy	NCT05319574	Zhao ZR, et al. 2023 (29).	II	Asian	46	IIA-IIIB(N2)	Durvalumab + Chemoradiotherapy	2	79.5	54.4	–
	NCT03694236	Hong MH, et al. 2023 (30).	II	Asian	24	III(Non-N3)	Durvalumab + Chemoradiotherapy	2	77.8	38.9	–
	NCT04245514	Bahce, I., et al. 2024 (31).	II	White	30	II-IIIB	Ipilimumab + Nivolumab + Chemoradiotherapy	2	63	50	–
Immunotherapy + Chemotherapy + Anti-angiogenesis	NCT05400070	Yan X, et al. 2023 (32).	II	Asian	39	II-IIIB	Sintilimab + Chemotherapy + Anlotinib	3	74.3	62.9	–
	NCT04846634	Wang C, et al. 2023 (33).	II	Asian	16	IIB-IIIB(N2)	Penpulimab + Chemotherapy + Anlotinib	4	70	50	–
	NCT06475755	Duan, H., et al. 2024 (34).	II	Asian	45	I-IIIB	Sintilimab + Chemotherapy + Anlotinib	3	66.7	57.8	81.5(64.5-90.9)
Immunotherapy + Anti-angiogenesis	NCT04846634	Wang C, et al. 2023 (33).	II	Asian	17	IIB-IIIB(N2)	Penpulimab + Anlotinib	4	80	60	–
	ChiCTR2000033588	Zhao J, et al. 2023 (35).	II	Asian	78	IIA-IIIB(only T3N2)	Camrelizumab + Apatinib	3	56.9	23.1	–
	NCT04040361	Aokage K, et al. 2023 (36).	II	Asian	24	IB-IIIA	Pembrolizumab + Ramucirumab	2	50	25	–
Immunotherapy + Immunotherapy	NCT03158129	Cascone T, et al. 2023 (37).	II	White (91%)/Black (4%)/Asian (5%)	21	I-IIIA	Nivolumab + Ipilimumab	3	38.1	28.6	–
	NCT03794544	Altorki NK, et al. 2021 (25).	II	White	21	IA3-IIIA	Durvalumab + Oleclumab	2	19	9.5	–
	NCT03794544	Altorki NK, et al. 2021 (25).	II	White	20	IA3-IIIA	Durvalumab + Monalizumab	2	30	10	–
	NCT03794544	Altorki NK, et al. 2021 (25).	II	White	16	IA3-IIIA	Durvalumab + Danvatirsen	4	31.3	12.5	–
	NCT04205552	Aigner C, et al. 2023 (38).	II	White	30	IB-IIIA	Nivolumab + Relatlimab	2	30	16.7	–
Immunotherapy + Chemotherapy	NCT03081689	Provencio, M., et al. 2019 (39).	II	White	46	IIIA	Nivolumab + Chemotherapy	3	76	54.3	–
	NCT04606303	Yan, S., et al. 2023 (40).	II	Asian	100	IIB-IIIB	Toripalimab + Chemotherapy	2-4	52	37	–
	NCT04304248	Zhao, Z., et al. 2021 (41).	II	Asian	33	IIIA-IIIB	Toripalimab + Chemotherapy	3	60.6	45.5	67.9(51.97-83.83)
	ChiCTR2100044645	Zhang, Y., et al. 2022 (42).	II	Asian	26	IIB-IIIB	Camrelizumab + Chemotherapy	2-4	38.4	19.2	–
	NCT04144608	Zhang, Y., et al. 2022 (43).	II	Asian	33	IIIA-IIIB	Toripalimab + Chemotherapy	2-4	45.5	33.3	72.9(57.73-88.07)
	NCT04326153	Sun, C., et al. 2023 (44).	II	Asian	30	IIIA-IIIB	Sintilimab + Chemotherapy	2-3	33.3	16.6	75(56-94)
	NCT02716038	Shu, C. A., et al. 2020 (45).	II	White	30	IB-IIIA	Atezolizumab + Chemotherapy	4	56.7	33.3	–
	NCT05024266	Shan, J., et al. 2024 (46).	II	Asian	35	IIIA-IIIB(N2)	Tislelizumab + Chemotherapy	2-4	68.6	40	61 (42.3-88)
	NCT06241807	Cai, G., et al. 2024 (47).	II	Asian	30	IIIA-IIIB(N2)	Camrelizumab + Chemotherapy	4	50	33.3	–

**Table 2:** Summary of randomized controlled trials evaluating neoadjuvant immunochemotherapy in NSCLC.

Registration Number	Study Phase	Author and Year	Patient Stage	Region/Race	Sample Size	Treatment	Therapy Cycles	MPR (%)	pCR (%)	24m EFS (%)
**NCT02998528**	III	Forde PM, et al. 2022 (4).	IB-IIIA	North American(22.9%)/European(22.9%)/Asian: (47.5%)/Other regions(6.7%)	179	Nivolumab + Chemotherapy	3	36.9	24	64(56.97-71.03)
				North American(27.9%)/European(14%)/Asian: (51.4%)/Other regions(6.7%)	179	Chemotherapy Alone	3	8.9	2.2	45(37.71-52.29)
**NCT03425643**	III	Wakelee H, et al. 2023 (5).	II-IIIB (N2)	Asian (31.2%)/Black (1.5%)/White (63%)/Missing data (3.3%)/Multiple (0.8%)	396	Pembrolizumab + Chemotherapy	4	30.2	18.1	62.4(56.8-67.5)
				Asian (31.3%)/Black (2.5%)/White (59.8%)/Missing data (4%)/Multiple (2.5%)	399	Chemotherapy + Placebo	4	11	4	40.6(34.8-46.3)
**NCT03800134**	III	Heymach JV, et al. 2023 (48).	II-IIIB (N2)	Asian (39.1%)/White (56.3%)/Other (4.6%)	366	Durvalumab + Chemotherapy	4	33.3	17.2	63.3(56.1-69.6)
				Asian (43.9%)/White (51.1%)/Other (5.1%)	374	Chemotherapy + Placebo	4	12.3	4.3	52.4(45.4-59)
**NCT04025879**	III	Cascone T, et al. 2024 (49).	II-IIIB (N2)	White (67.7%)/Black (1.7%)/Asian (28.8%)/Other (1.7%)	229	Nivolumab + Chemotherapy	4	35.4	25.3	
				White (75.4%)/Black (1.7%)/Asian (21.6%)/Other (1.3%)	232	Chemotherapy + Placebo	4	12.1	4.7	
**NCT04379635**	III	Yue D, et al. 2023 (50).	II-IIIA	Asian	226	Tislelizumab + Chemotherapy	3-4	56.2	40.7	–
					227	Chemotherapy + Placebo	3-4	15	5.7	–
**NCT04158440**	III	Lu S, et al. 2024 (6).	III	Asian	202	Toripalimab + Chemotherapy	3	48.5	24.8	64.71(58.11-71.29)
					202	Chemotherapy + Placebo	3	8.4	1	38.7(31.98-45.42)
**NCT04338620**	II	J Lei, et al. 2023 (51).	IIIA-IIIB (T3N2)	Asian	43	Camrelizumab + Chemotherapy	3	65.1	32.6	76.9(56.3-88.7)
					45	Chemotherapy Alone	3	15.6	8.9	67.6(48.0-81.2)
**NCT03838159**	II	Provencio M, et al. 2023 (52).	IIIA-IIIB	White	57	Nivolumab + Chemotherapy	3	53	37	67(54.79 - 79.21)
					29	Chemotherapy Alone	3	14	7	35(17.64 - 52.36)

### Characteristics of included studies

A total of 32 single-arm studies with 1,208 patients (stages I to IIIB) evaluated various neoadjuvant ICI-based therapies for resectable NSCLC, including ICIs alone or combined with chemotherapy, radiotherapy, chemoradiotherapy, anti-angiogenesis agents, or dual immunotherapy. Treatment cycles ranged from 1 to 4, with MPR rates from 6.7% to 80% and pCR rates from 0% to 62.9%. Combinations with chemoradiotherapy or anti-angiogenesis agents showed relatively higher response rates.

Eight RCTs with 3,385 patients primarily compared ICIs plus chemotherapy to chemotherapy-based controls. Treatment cycles were typically fixed at 3 or 4, with MPR rates ranging from 30.2% to 65.1% and pCR rates from 17.2% to 40.7%. Control arms reported MPR rates from 8.4% to 15.6% and pCR rates from 1% to 8.9%. The studies focused on stages IB to IIIB, with an emphasis on stage III.

### Study quality assessment

To assess publication bias, we compared the effect size distributions between single-arm studies and RCTs. The Mann-Whitney U test showed no significant difference (p = 0.2334), suggesting minimal publication bias. Density plots confirmed the overlap in effect size distributions (**Supplementary Figure S1**). RCTs showed good randomization, blinding, and data completeness, but some limitations in allocation concealment and selective reporting bias (**Supplementary Figure S2**). Single-arm studies had lower scores in sample size adequacy and confounding control, but performed well in outcome clarity and follow-up completeness, indicating overall reliability (**Supplementary Figure S3**).

### Efficacy of immunotherapy combinations in single-arm studies

**Figure 2:**
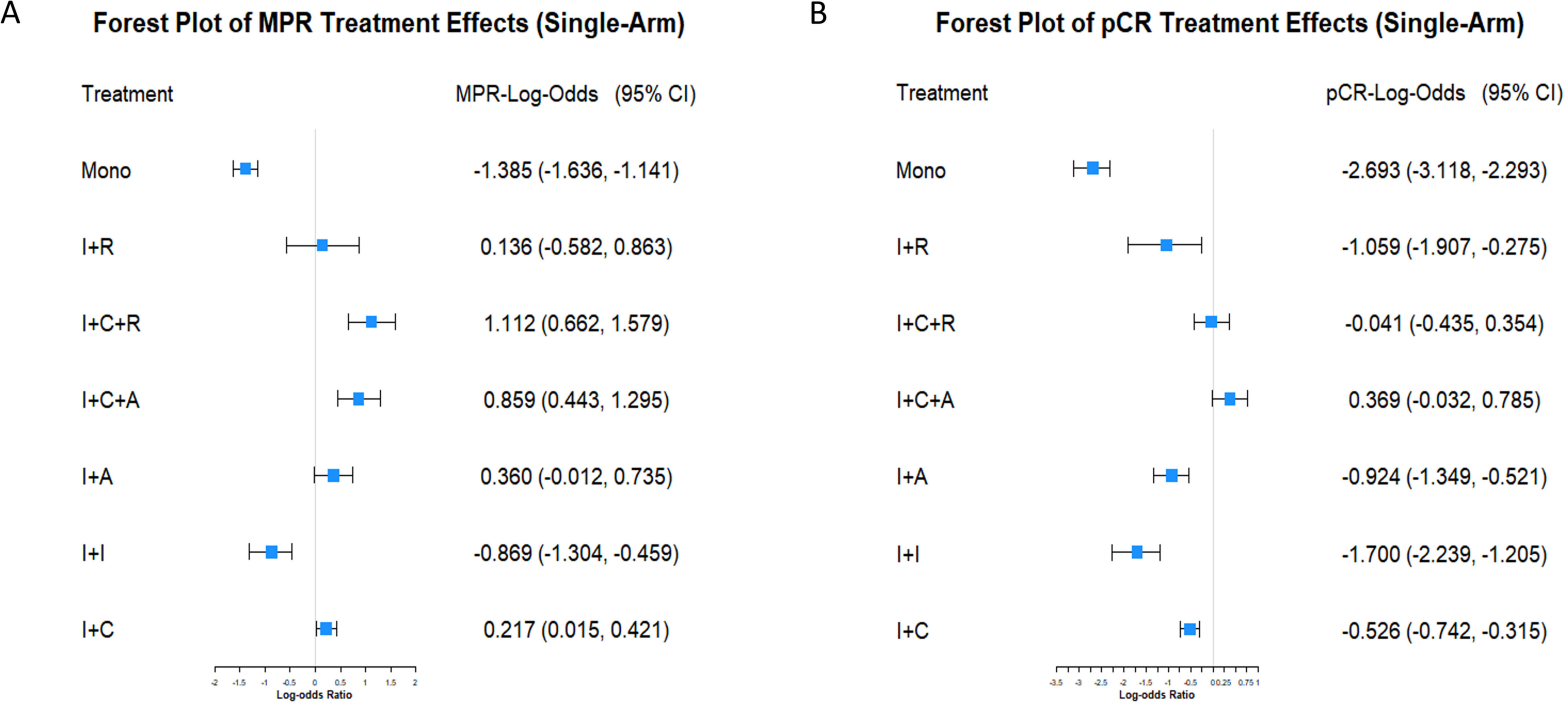
Forest plots of MPR and pCR treatment effects in single-arm studies. **(A)** Forest plot displaying the LogOR and 95% CI for MPR among various neoadjuvant immunotherapy strategies. Treatments include immunotherapy monotherapy (Mono), immunotherapy combined with radiotherapy (I+R), chemotherapy (I+C), anti-angiogenesis therapy (I+A), dual immunotherapy (I+I), immunotherapy combined with chemotherapy and radiotherapy (I+C+R), and chemotherapy combined with anti-angiogenesis therapy (I+C+A). **(B)** Forest plot showing the LogOR and 95% CI for pCR under the same treatment strategies.

MPR efficacy of seven immunotherapy strategies was analyzed from 32 single-arm studies using a Bayesian hierarchical model. Monotherapy showed the lowest efficacy (posterior mean: -1.384, 95% CI: -1.640, -1.141), while immunotherapy combined with chemoradiotherapy achieved the highest efficacy (posterior mean: 1.110, 95% CI: 0.663, 1.576). Other combinations, like immunotherapy with anti-angiogenesis and chemotherapy, also showed moderate efficacy (posterior mean: 0.858, 95% CI: 0.438, 1.295) (**Figure 2A**). For pCR, while monotherapy again showed the lowest efficacy, certain combination strategies (e.g., immunotherapy combined with anti-angiogenesis and chemotherapy) exhibited significant efficacy in MPR but failed to achieve statistical significance in pCR (posterior mean: 0.370, 95% CI: -0.025, 0.773) (**Figure 2B**). Posterior density plots (**Supplementary Figure S4**) and model diagnostics (**Supplementary Figure S5**) confirmed the reliability of the results, with all PSRF values equal to 1.00.

### Comparative efficacy of neoadjuvant immunotherapy combinations: weighted Bayesian meta-analysis of single-arm studies and RCTs

**Figure 3:**
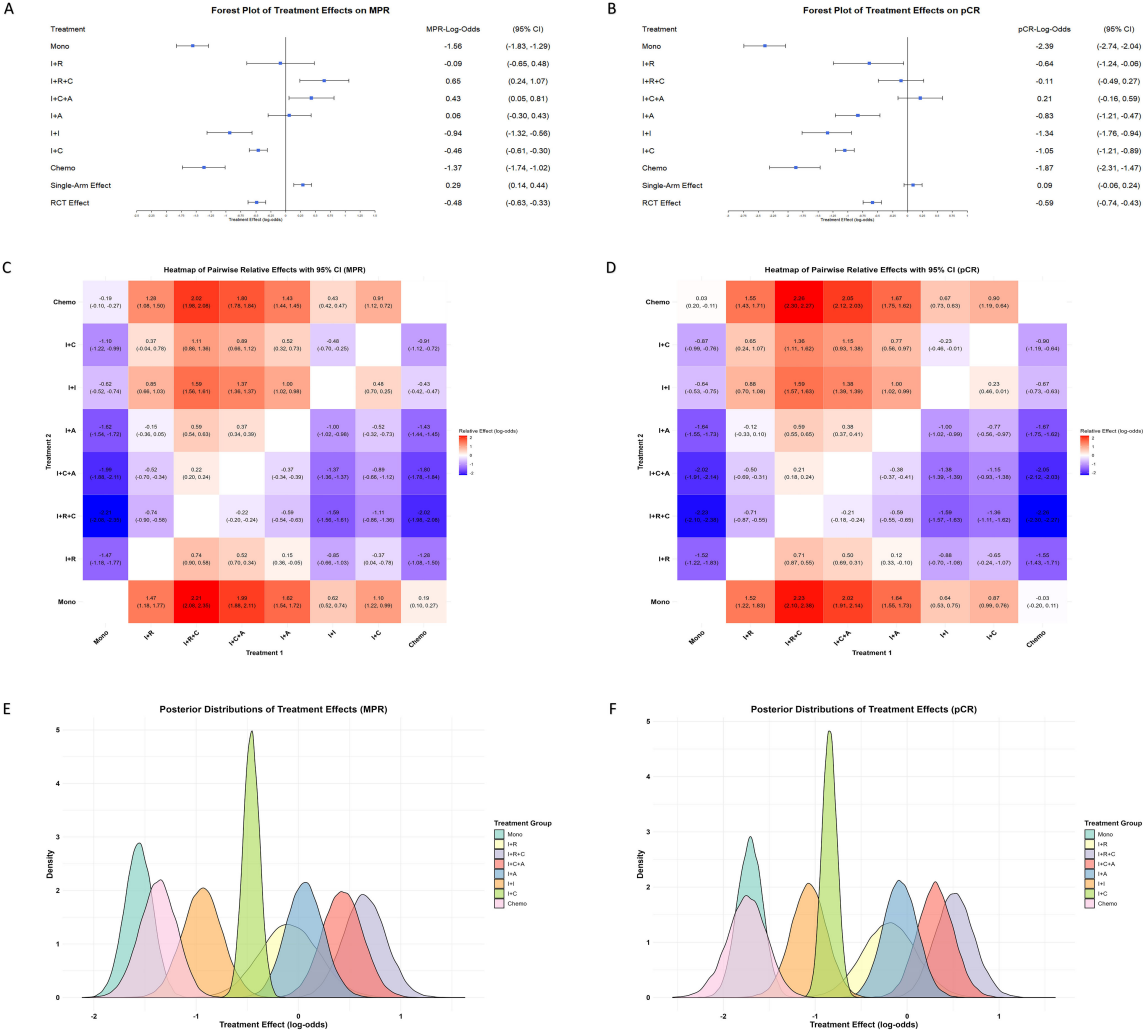
Comparison of treatment effects on MPR and pCR across single-arm and RCTs. **(A)** Forest plot of treatment effects on MPR, presented as LogOR with 95% CI, across various neoadjuvant strategies in both single-arm and RCTs. Treatments include immunotherapy monotherapy (Mono), immunotherapy combined with radiotherapy (I+R), chemotherapy (I+C), anti-angiogenesis therapy (I+A), dual immunotherapy (I+I), and immunotherapy with chemotherapy and radiotherapy (I+C+R), chemotherapy and anti-angiogenesis therapy (I+C+A). **(B)** Forest plot of treatment effects on pCR under the same treatment strategies. Heatmaps illustrating pairwise comparisons of treatment effects on MPR **(C)** and pCR **(D)**, showing LogOR values for each treatment combination. Warmer colors (red) represent higher positive LogOR values, indicating stronger effects, while cooler colors (blue) denote lower effects. Probability density plots of treatment effect distributions for MPR **(E)** and pCR **(F)**, highlighting variability and overlapping trends among the treatment strategies. Peaks and widths of distributions reflect the central tendency and uncertainty for each treatment.

Based on a weighted Bayesian model analysis of data from single-arm studies and RCTs, significant differences were identified among neoadjuvant treatment strategies for improving pCR and MPR in NSCLC (**Figures 3A, B**). Immunotherapy monotherapy was used as the reference group, and all effect estimates reflect relative efficacy compared to it. The results showed that immunotherapy combined with chemotherapy and anti-angiogenesis was the most effective strategy, with pCR and MPR effect estimates of 0.21 (95% CI: -0.16, 0.59) and 0.43 (95% CI: 0.05, 0.81), respectively. Immunotherapy combined with chemoradiotherapy showed the strongest effect on MPR at 0.65 (95% CI: 0.24, 1.07) and ranked second for pCR. In contrast, monotherapy and chemotherapy alone were the least effective, highlighting the significant advantages of multimodal combination therapies and their potential to optimize treatment strategies for NSCLC. Comparing single-arm studies and RCTs revealed that single-arm studies reported higher effect estimates, with pCR and MPR values of 0.09 (95% CI: -0.06, 0.24) and 0.29 (95% CI: 0.14, 0.44), potentially reflecting design biases. In contrast, RCTs provided more conservative estimates, suggesting their rigor yields more realistic treatment effects.

The pairwise relative effect heatmaps further highlight the comparative advantages of multimodal therapies, with combination treatments consistently outperforming single-modality treatments (**Figures 3C, D**). The posterior distributions of treatment effects provide insights into the precision of the effect estimates (**Figures 3E, F**). The narrower posterior distributions observed for treatments such as immunotherapy combined with chemotherapy highlight the stability and precision of their effect estimates. However, superior effect sizes observed in immunotherapy combined with chemotherapy and anti-angiogenesis and immunotherapy combined with chemoradiotherapy further reinforce the potential of multimodal therapies to achieve optimal outcomes.

### No further improvement in efficacy beyond three treatment cycles

The Bayesian hierarchical model evaluated the efficacy of different neoadjuvant chemoimmunotherapy cycles (3 cycles, mixed cycles, and 4 cycles) on MPR and pCR. Mixed cycles included combinations such as 2–3, 2–4, and 3–4 cycles (**Supplementary Figure S6**).

**Figure 4:**
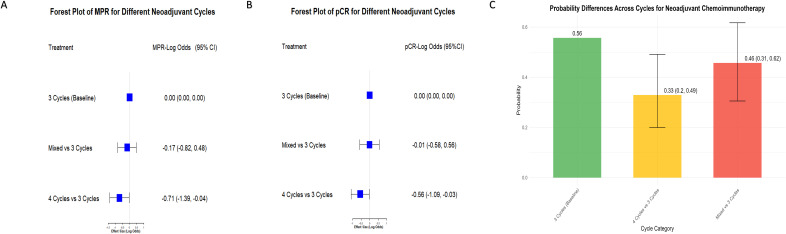
Comparison of MPR and pCR Across different neoadjuvant immunochemotherapy cycles. **(A)** Forest plot of MPR treatment effects comparing different neoadjuvant immunochemotherapy cycles. The baseline is set as 3 cycles, with comparisons made against mixed cycles (including combinations such as 2–3, 2–4, and 3–4 cycles) and 4 cycles. Results are shown as LogOR with 95% CI. Negative LogOR values indicate reduced likelihood of achieving MPR compared to 3 cycles. **(B)** Forest plot of pCR treatment effects comparing 3 cycles (baseline) with mixed cycles (2–3, 2–4, and 3–4 cycles) and 4 cycles. LogOR with 95% CI illustrate differences in pCR rates between groups. **(C)** Bar chart showing the proportion of MPR for different neoadjuvant cycle groups (3 cycles, mixed cycles including 2–3, 2–4, and 3–4 cycles, and 4 cycles). Error bars represent the 95% CI, highlighting variability in MPR rates among the groups.

The MPR forest plot (**Figure 4A**) showed that 3 cycles were significantly more effective than 4 cycles, with an effect size of -0.71 (95% CI: -1.39, -0.04), while mixed cycles showed no significant difference (-0.17, 95% CI: -0.82, 0.48). Similarly, the pCR forest plot (**Figure 4B**) confirmed the advantage of 3 cycles over 4 cycles (-0.56, 95% CI: -1.09, -0.03), with no significant difference for mixed cycles (-0.01, 95% CI: -0.58, 0.56).

The probability plot (**Figure 4C**) further highlighted that 3 cycles had the highest probability of success (0.56, 95% CI: 0.49, 0.62) compared to mixed cycles (0.46) and 4 cycles (0.33). These findings suggest that increasing the number of cycles beyond 3 provides no additional benefit and may even reduce efficacy. Convergence diagnostics confirmed the reliability of the analysis, with all PSRF equal to 1.00.

### No racial or ICI-related differences in MPR and pCR outcomes

**Figure 5:**
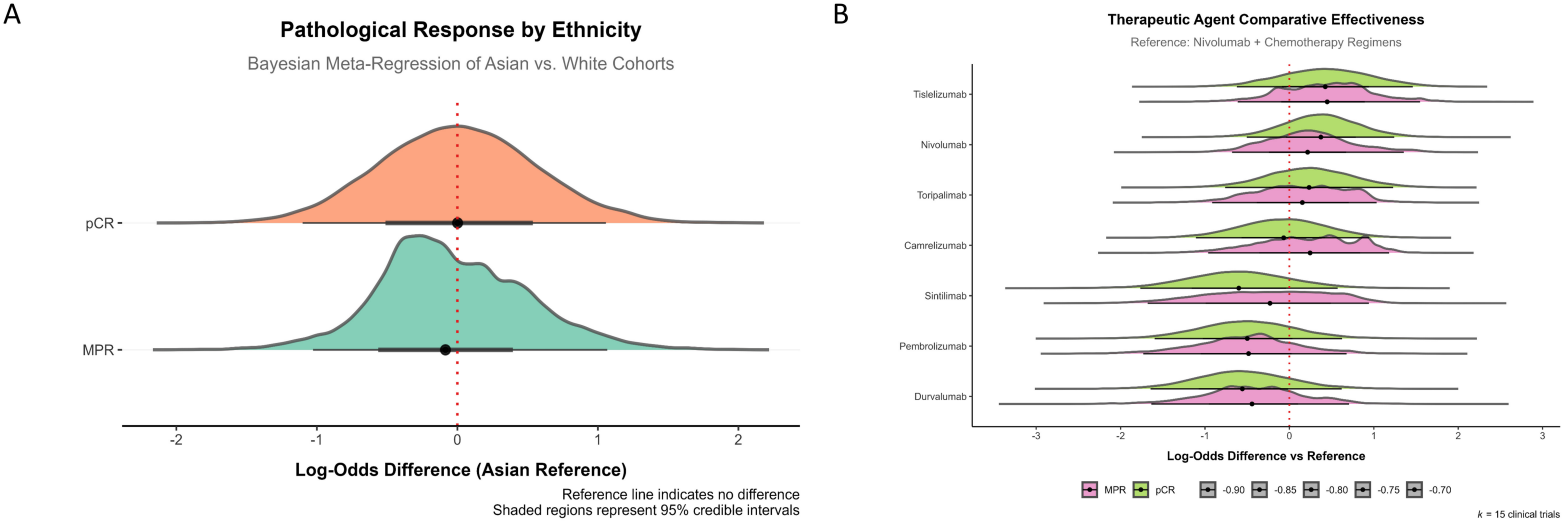
Ethnic and ICIs effects on pathological response in chemoimmunotherapy. **(A)** Pathological response by ethnicity, showing Bayesian meta-regression of Asian versus White cohorts. The comparison of log-odds difference between the two ethnic groups for MPR and pCR demonstrates no significant differences, as indicated by the reference line at zero. **(B)** Therapeutic agent comparative effectiveness for various ICIs. The data shows the log-odds difference relative to the reference (Nivolumab + chemotherapy regimens). No significant differences were observed in MPR or pCR efficacy among the different ICIs.

The effects of race (White vs. Asian) and different ICIs on MPR and pCR in chemoimmunotherapy were further investigated, as these two racial groups constituted the majority of the study population (**Table 1**, **Supplementary Table S2**). The results revealed no significant differences in MPR and pCR between the White and Asian groups. The median difference for MPR was -0.08 (95% CI: [−1.02, 1.08]) and for pCR was 0.00 (95% CI: [−1.06, 1.10]) (**Figure 5A**). Although variations in efficacy were observed, no statistically significant differences in MPR and pCR were found among the ICIs (**Figure 5B**).

### MPR and pCR as predictors of EFS in chemoimmunotherapy

We investigated the association between MPR and pCR with EFS to evaluate their prognostic predictive value. This study analyzed data from all treatment groups receiving neoadjuvant immunotherapy combined with chemotherapy, of which 5 RCTs and 2 single-arm studies reported EFS data stratified by MPR or pCR (**Table 3**).

**Table 3:** Summary of studies evaluating the association between MPR/pCR and EFS in neoadjuvant immunochemotherapy for NSCLC.

Registration Number	Study Type	Neo-Treat	Sample Size	MPR (%)	24m EFS (MPR) (%)	24m EFS (non-MPR) (%)	pCR (%)	24m EFS (pCR) (%)	24m EFS (non-pCR) (%)	HR (MPR vs. Non-MPR, EFS, 95% CI)	HR (pCR vs. Non-pCR, EFS, 95% CI)
NCT02998528	RCT	Nivolumab + Chemotherapy	179	36.9	N/A	N/A	24	94.5	51.5	N/A	0.13 (0.05–0.37)
NCT03425643	RCT	Pembrolizumab + Chemotherapy	396	30.2	92.3	48.7	18.1	97.1	56.1	0.165 (0.087–0.313)	0.178 (0.079–0.402)
NCT04025879	RCT	Nivolumab + Chemotherapy	229	35.4	85.1	52.3	25.3	84.8	53.8	0.3 (0.16–0.57)	0.29 (0.13–0.62)
NCT04158440	RCT	Toripalimab + Chemotherapy	202	48.5	79.1	44.5	24.8	92	56	0.24 (0.13–0.45)	0.22 (0.09–0.55)
NCT03838159	RCT	Nivolumab + Chemotherapy	57	53	N/A	N/A	37	100	49.5	N/A	0.091 (0.012–0.694)
NCT04304248	Single Arm	Toripalimab + Chemotherapy	33	60.6	86	43	45.5	N/A	N/A	0.1 (0.01–0.9)	N/A
NCT04326153	Single Arm	Sintilimab + Chemotherapy	30	33.3	100	29	16.6	N/A	N/A	0.022 (0.009–0.054)	N/A

**Figure 6:**
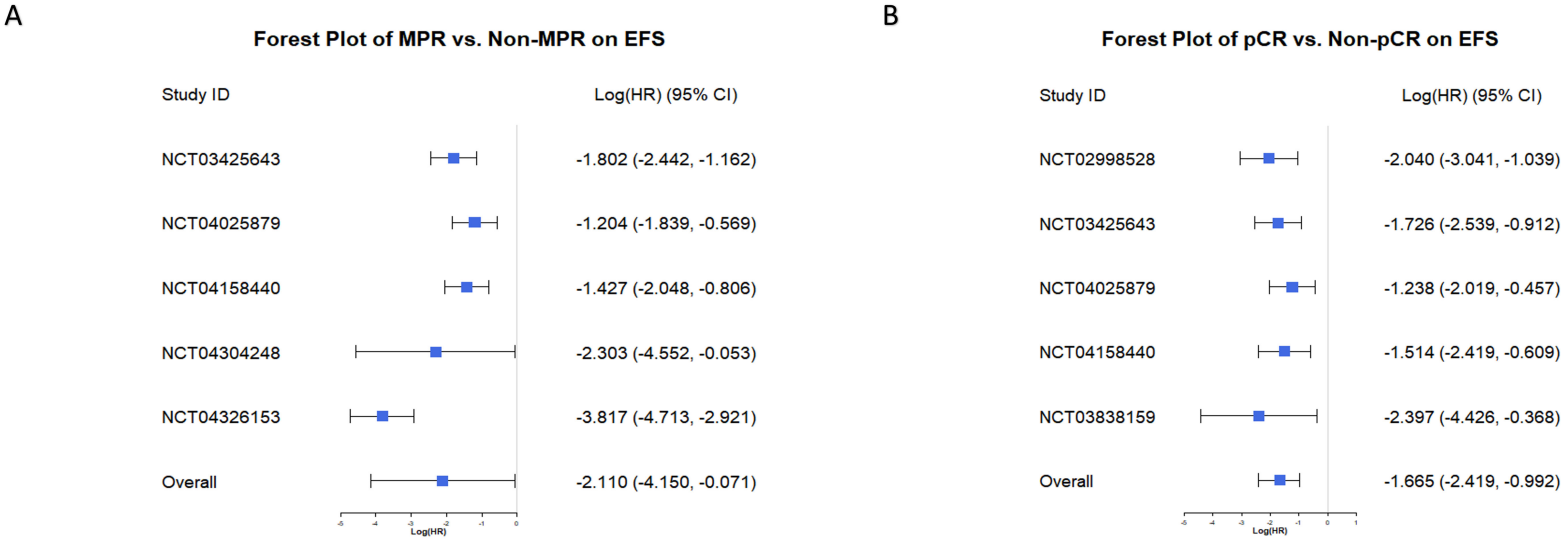
Forest plots of MPR and pCR predicting Event-Free Survival (EFS). **(A)** Forest plot of the effect of MPR versus non-MPR on EFS. Log (HR) and 95% CI are displayed for individual studies and the overall effect. Negative Log (HR) values indicate improved EFS in the MPR group compared to the non-MPR group. **(B)** Forest plot of the effect of pCR versus non-pCR on EFS. Log (HR) and 95% CI are shown for each study and the combined analysis. Negative Log (HR) values indicate better EFS in the pCR group compared to the non-pCR group.

The Bayesian random-effects model demonstrated that all individual studies consistently showed a protective effect of achieving MPR or pCR on EFS, with logHR values below 0. For MPR (**Figure 6A**), the overall logHR was -2.110 (95% CI: -4.150, -0.071), and for pCR (**Figure 6B**), it was -1.665 (95% CI: -2.419, -0.992). These results confirm that achieving MPR or pCR significantly reduces event risk, highlighting their strong predictive value for EFS. The consistent findings across studies further underscore their reliability as key indicators for evaluating neoadjuvant chemoimmunotherapy efficacy in NSCLC.

Using a sample-size-weighted linear regression model, we evaluated the potential of MPR and pCR LogOR as surrogate markers for EFS LogHR. MPR LogOR was significantly negatively correlated with EFS LogHR (β = -0.6836, 95% CI: -1.606 to -0.319, P = 0.027), with an R² of 0.845, indicating that MPR LogOR explained 84.5% of the variance in EFS LogHR. In contrast, pCR LogOR showed a weaker negative correlation (β = -0.3470, 95% CI: -0.677 to 0.028, P = 0.078) and a lower R² of 0.698 (69.8% of the variance explained).

**Figure 7:**
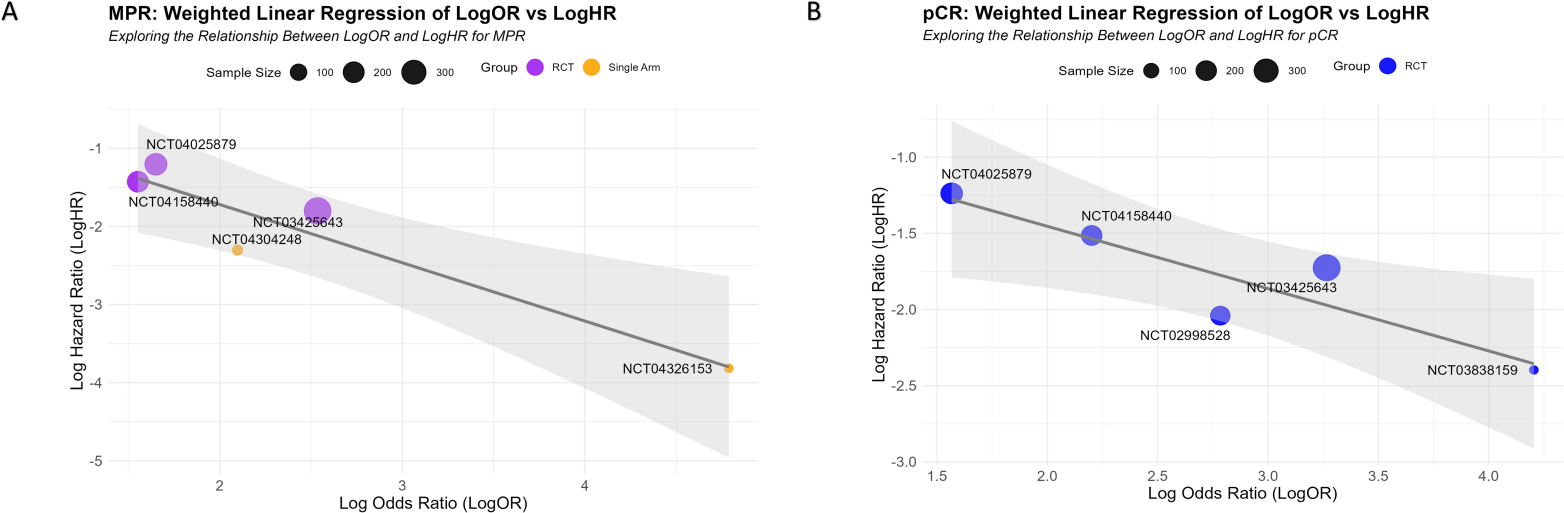
Correlation between MPR and pCR LogOR and EFS LogHR. **(A, B)** Scatter plots showing the relationships between MPR LogOR **(A)** and pCR LogOR **(B)** with EFS LogHR. Each circle represents an individual study, with the size of the circle proportional to the study’s sample size. The fitted regression lines illustrate the negative correlations between LogOR and LogHR, with the gray shaded areas representing the 95% CI.

Bootstrap analysis confirmed the robustness of the models: for MPR, the 95% CI for β and R² were -1.606 to -0.319 and 0.377 to 1.000, respectively, while for pCR, they were -0.677 to 0.028 and 0.001 to 1.000. Scatter plots with fitted curves illustrated that as MPR or pCR LogOR increased, EFS LogHR decreased, with MPR showing a stronger correlation (**Figures 7A, B**). These findings highlight the superior predictive capability of MPR LogOR over pCR LogOR for EFS LogHR at the trial level.

## Discussion

Monotherapy with chemotherapy or immunotherapy shows low MPR and pCR rates in neoadjuvant treatment, suggesting these strategies alone are inadequate. Although several phase III RCTs indicate that neoadjuvant immunochemotherapy improves MPR and pCR, it is not necessarily the most effective approach. Immunochemotherapy combined with anti-angiogenesis or radiotherapy both yield MPR rates above 60%, suggesting superior response rates. Bayesian hierarchical model analysis of single-arm studies and RCTs consistently showed that these two combinations achieved the best MPR and pCR outcomes.

Immunochemotherapy combined with anti-angiogenesis works by improving tumor vascularization, reducing immunosuppressive factors within the tumor microenvironment, and enhancing immune cell infiltration, thereby augmenting the therapeutic response (8). On the other hand, immunochemotherapy combined with radiotherapy enhances antitumor efficacy through local tumor cell apoptosis and activation of local immune responses (9). However, despite these promising early results indicating favorable efficacy and manageable safety, uncertainties remain, particularly due to the predominance of single-arm studies with small sample sizes, and the lack of large-scale, multi-center validation. Therefore, further rigorous randomized controlled trials are necessary to evaluate the long-term efficacy, safety, and patient tolerability of these treatment combinations.

Notably, Single-arm studies show differing effects of immune combination anti-angiogenesis therapy and chemotherapy on MPR and pCR, likely due to biological mechanisms and data heterogeneity. MPR and pCR reflect different levels of pathological responses, and achieving MPR does not guarantee pCR. Chemotherapy plus immunotherapy reduces tumor burden and boosts immune responses, improving short-term MPR. However, bone marrow suppression may later reduce immune cell numbers, affecting pCR. Differences in study design, patient population, treatment cycles, and drug use may explain inconsistencies in MPR and pCR results. Interestingly, the consistent effects of immune combination chemotherapy and anti-angiogenesis therapy suggest that combination treatments enhance therapeutic stability via synergistic mechanisms.

The optimal number of treatment cycles for neoadjuvant immunochemotherapy in NSCLC is still debated. In this meta-analysis, combining data from RCTs and single-arm studies, we found that three cycles of immunochemotherapy produced the best outcomes. Extending treatment to four cycles did not improve MPR and pCR, and even slightly reduced them. These findings align with some existing studies. For example, the neoSCORE study showed that three cycles of neoadjuvant immunochemotherapy resulted in a higher MPR rate compared to two cycles, but the difference was not statistically significant (10). Additionally, the study by Deng et al. found that three and four cycles of neoadjuvant immunochemotherapy may result in higher MPR rates compared to two cycles in stage III NSCLC (11). Zhang et al. pointed out that extending the treatment cycle may improve surgical safety, but the MPR rate may not increase significantly (12). Insufficient treatment duration may fail to fully activate tumor-specific T cells, while excessive treatment may lead to T cell dysfunction or exhaustion (13). The optimal therapeutic effect can only be achieved if primary tumors are resected at the peak of tumor-specific T cell expansion. Furthermore, extending the treatment cycle may increase drug-related adverse events and may result in missing the optimal surgical timing.

Although studies show racial differences in immune characteristics (14, 15), research on racial disparities in immunotherapy outcomes is limited. One study found longer survival in Asian NSCLC patients treated with atezolizumab (16), while another compared immunotherapy efficacy between Asian and White patients with resectable NSCLC without assessing benefit differences (17). Our study found no significant difference in MPR and pCR between Asian and White patients receiving immune combination chemotherapy, similar to breast cancer findings (18), suggesting race is not a major factor in immunotherapy outcomes. MPR and pCR are short-term indicators, and long-term efficacy requires further follow-up. While some studies rank different ICIs through network meta-analysis (19), our research found no significant differences in MPR and pCR, indicating that ICI type may not be the main determinant of efficacy, which is influenced by factors like treatment protocol, patient status, and tumor staging.

MPR and pCR are being explored as potential predictors of EFS in neoadjuvant immunochemotherapy for NSCLC. This study used EFS data stratified by MPR and pCR status (based on KM plots) to further validate their predictive value. Compared with previous studies that analyzed the overall MPR and pCR proportions in relation to overall EFS (20), our research directly used subgroup data, providing a more accurate reflection of the predictive efficacy of MPR and pCR for EFS. These findings are consistent with the literature. For example, the CheckMate 816 trial demonstrated that patients who achieved pCR after chemoimmunotherapy had significantly improved EFS (HR=0.13, 95% CI: 0.05–0.37) (4). Our analysis showed that MPR outperforms pCR in predicting EFS, as it better reflects overall tumor regression and captures partial pathological responses. While both MPR and pCR are strongly correlated with EFS at the patient level, challenges exist in using them as surrogate endpoints for EFS in trials. Variability in pathological evaluation, postoperative treatment differences, and timing issues may limit their broader applicability and impact their correlation with long-term survival. However, targeted subgroup analyses and a larger sample size underscore the value of MPR and pCR in predicting EFS, offering a reliable basis for evaluating neoadjuvant therapy efficacy.

## Limitations

There are limitations to this systematic review and meta-analysis. The design limitations of some single-arm studies may have led to overestimation of efficacy, although the Bayesian model mitigated this bias to some extent. Variability in pathological evaluation standards and postoperative treatments across studies may affect the generalizability of the findings. Additionally, the short follow-up duration limits the validation of long-term outcomes, such as OS. This study primarily relied on aggregated data and lacked individual patient data, which restricts deeper analyses of subgroups and potential influencing factors.

## Conclusions

This study evaluated the efficacy of neoadjuvant immunotherapy strategies for resectable NSCLC, suggesting that immunochemotherapy combined with anti-angiogenesis agents may be a highly effective approach. Three cycles of neoadjuvant immunochemotherapy were identified as a potentially optimal treatment duration. Additionally, MPR and pCR were confirmed as useful predictors of EFS, with MPR demonstrating greater predictive capability. These findings offer important evidence and insights for optimizing neoadjuvant treatment strategies.

## Data Availability

The original contributions presented in the study are included in the article/**Supplementary Material**. Further inquiries can be directed to the corresponding author.
