# Supplementary material for: Neoadjuvant immunotherapy for NSCLC: superior combination strategies, optimal treatment cycles, and predictive indicators from a Bayesian meta-analysis
Source: Front Immunol. 2025 Mar 27;16:1548665. doi: 10.3389/fimmu.2025.1548665 (PMC11983614; doi:10.3389/fimmu.2025.1548665)
Supplement: Supplementary file 1 [file DataSheet1.docx]

**Supplementary**

**Figure S1.** Distribution Comparison of Effect Sizes between Single-Arm Studies and Randomized Controlled Trials (RCTs).

**Figure S2.** Risk of bias assessment for RCTs.

**Figure S3.** Newcastle-Ottawa Scale (NOS) for Single-Arm Studies

Chemotherapy.

**Figure S4.** Posterior Distributions of MPR and pCR Estimates for Different Neoadjuvant Treatments in Single-Arm Studies.

**Figure S5.** Trace Plots for MPR and pCR Estimates Across Neoadjuvant Treatments.

**Figure S6.** Summary of Different Treatment Cycles and MPR/pCR Outcomes in Neoadjuvant Chemoimmunotherapy Studies.

**Table S1.** Search strategy

**Table S2.** Summary of Racial Distribution in Studies on Immunotherapy Checkpoint Inhibitors Combined with Chemotherapy

**
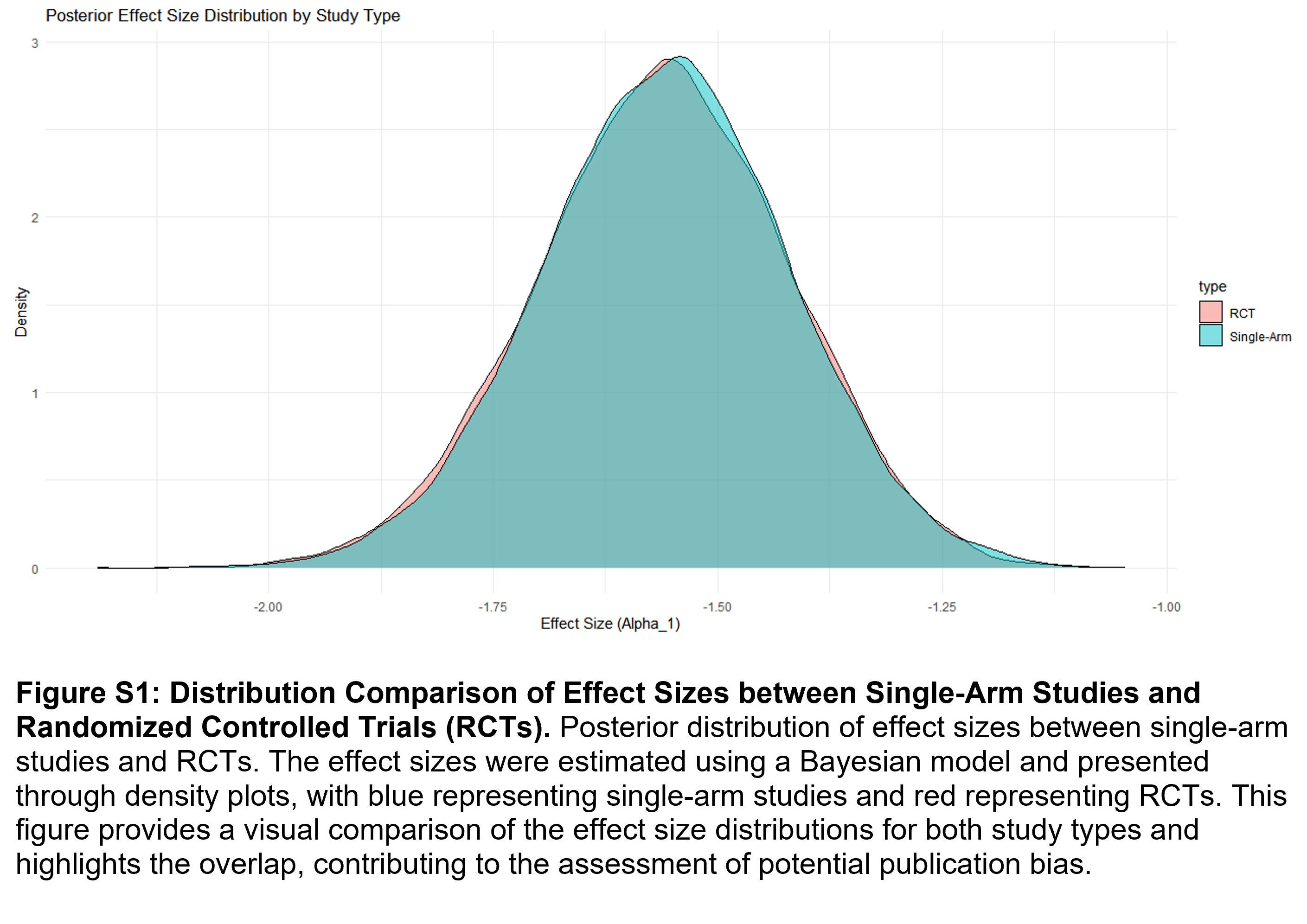
**

**
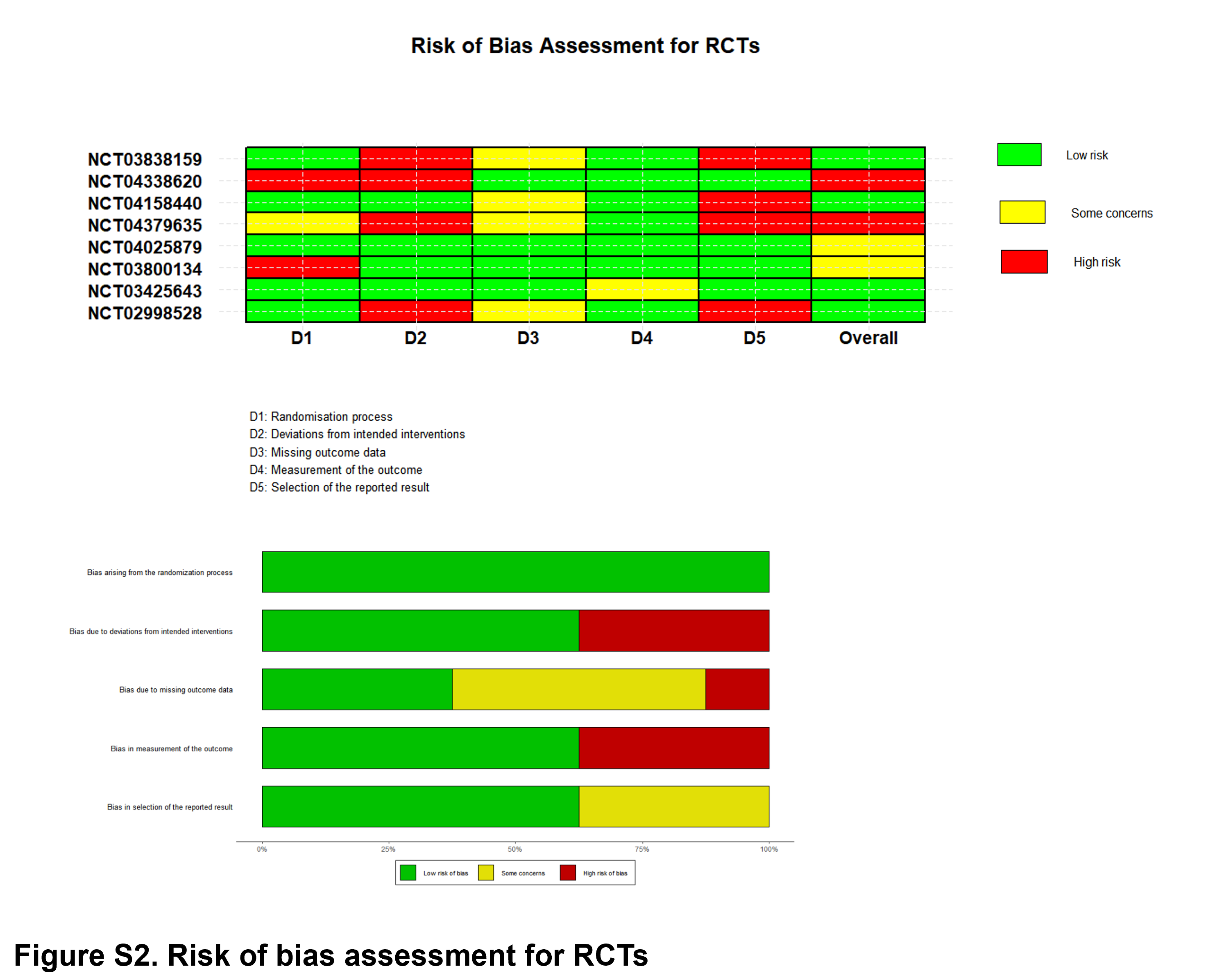
**

**
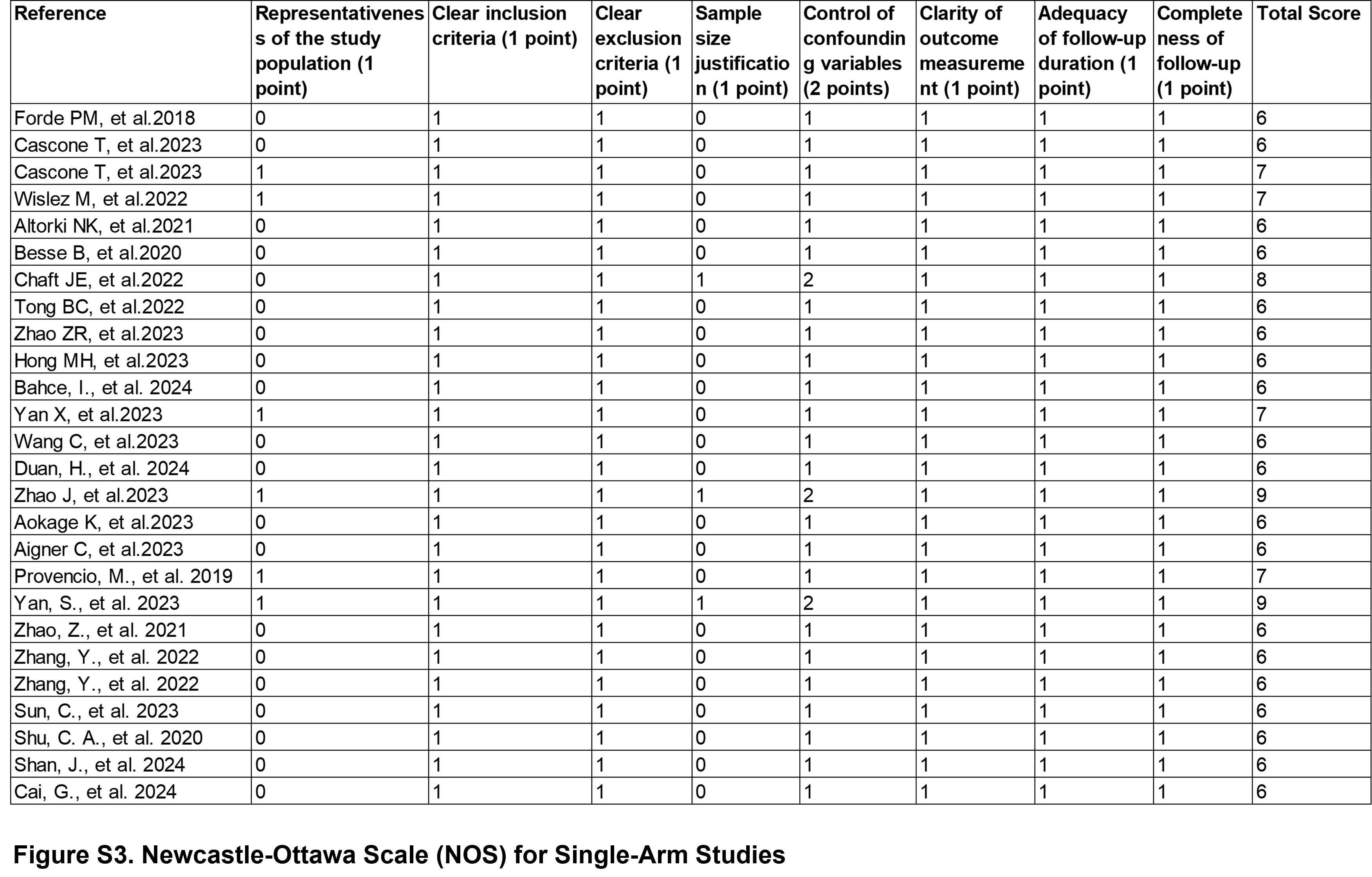
**

**
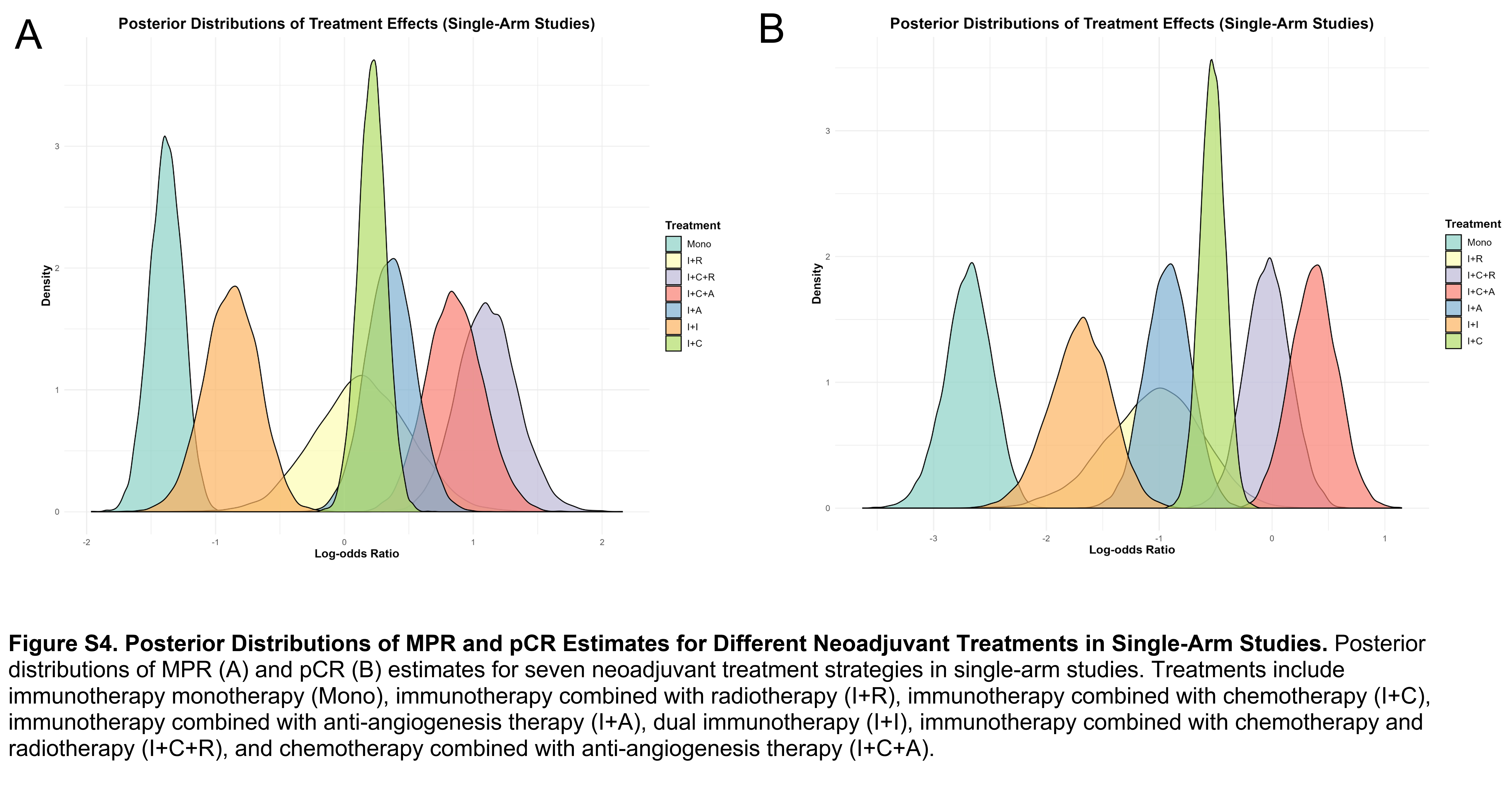
**

**
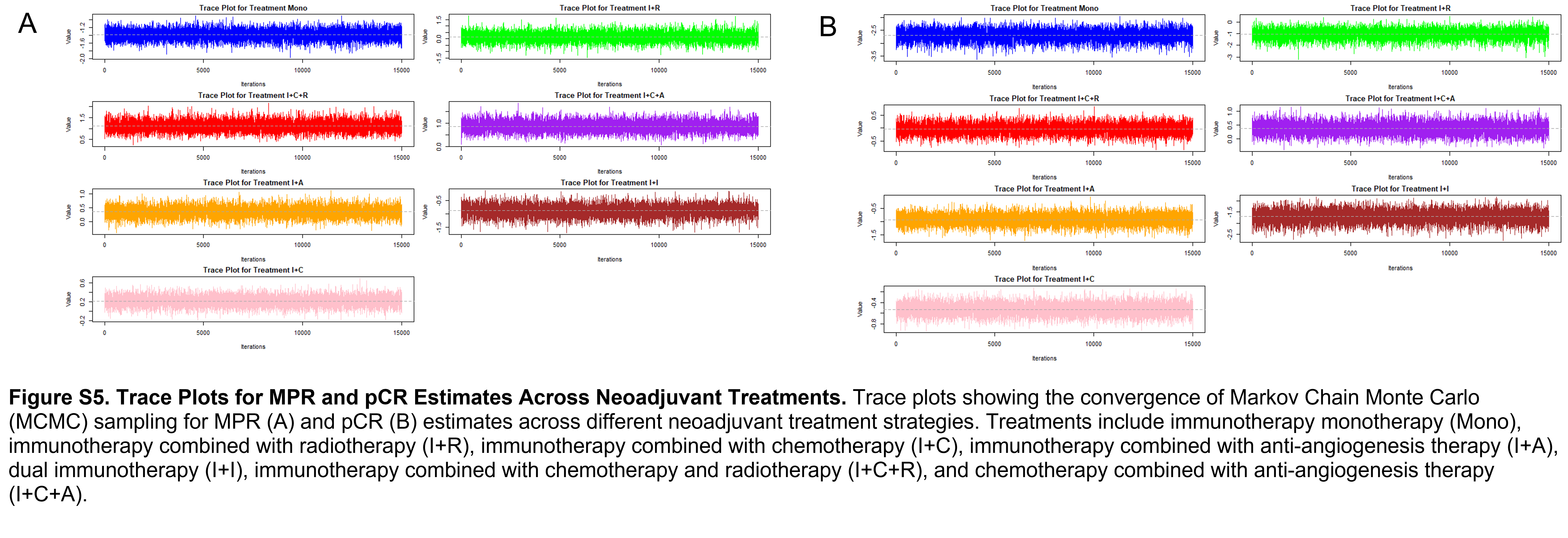
**

Figure S6. Summary of Different Treatment Cycles and MPR/pCR Outcomes in Neoadjuvant Chemoimmunotherapy Studies.

**Table S1. Search strategy**

| **PubMed search query** | 649 |
| --- | --- |
| ((non small cell* OR nonsmall cell* OR non-small cell* OR large cell* OR squamous cell* OR squamous small cell* OR nonsquamous cell* OR non-squamous cell* OR nonsquamous small cell* OR non-squamous small cell* OR epidermoid) AND (cancer* OR carcin* OR tumor* OR tumour* OR neoplas* OR oncol* OR malignan*)) AND (lung* OR pulmonary OR bronchial OR bronchus) OR ((lung* OR pulmonary OR bronchial) AND (adenocarcin* OR adenocancer*)) OR NSCLC* AND (neoadjuvant OR neo-adjuvant OR perioperative OR preoperative OR induction therapy) AND (immunotherapy OR immunotherapy monotherapy OR immunotherapy combination OR chemoimmunotherapy OR chemo-immunotherapy OR chemotherapy AND immunotherapy OR radiotherapy OR stereotactic body radiation therapy OR sequential immunochemotherapy OR anti-angiogenesis OR combination therapy OR dual immunotherapy OR chemotherapy OR Chemoradiotherapy) AND (nivolumab OR pembrolizumab OR durvalumab OR atezolizumab OR sintilimab OR camrelizumab OR penpulimab OR anlotinib OR apatinib OR ramucirumab OR oleclumab OR monalizumab OR danvatirsen OR relatlimab OR ipilimumab OR tislelizumab OR toripalimab OR immune checkpoint inhibitors OR immunotherapy agents OR PD-1 inhibitor OR PD-L1 inhibitor) NOT (Animals NOT Humans) NOT （news OR comment OR letter OR editorial OR case report*） | |
| **EMBASE search query** | 467 |
| (‘nivolumab’/exp OR ‘pembrolizumab’/exp OR ‘durvalumab’/exp OR ‘atezolizumab’/exp OR ‘camrelizumab’:ab,ti OR ‘tislelizumab’:ab,ti OR ‘sintilimab’:ab,ti OR ‘toripalimab’:ab,ti OR ‘relatlimab’:ab,ti OR ‘ipilimumab’:ab,ti OR ‘immune checkpoint inhibitor’:ab,ti OR ‘immunotherapy’:ab,ti OR ‘immunotherapy agents’:ab,ti OR ‘PD-1 inhibitor’:ab,ti OR ‘PD-L1 inhibitor’:ab,ti OR ‘immune chemotherapy’:ab,ti) AND (‘perioperative’:ab,ti OR ‘neoadjuvant’:ab,ti OR ‘adjuvant’:ab,ti) AND ('non small cell lung cancer'/exp OR 'NSCLC':ab,ti OR 'non-small-cell':ab,ti) AND (clinical trial OR randomized controlled trial) NOT (animals NOT humans) NOT (news OR comment OR letter OR editorial OR case report* OR review OR meta OR preclinical study OR retrospective study OR non-peer-reviewed) | |
| **Web of Science search query** | 2860 |
| (((((TS=(‘non small cell lung cancer’ OR ‘NSCLC’ OR ‘non-small-cell lung cancer’ OR ‘non-small cell’ OR ‘non-small-cell’ OR ‘large cell lung cancer’ OR ‘squamous cell lung cancer’ OR ‘adenocarcinoma’)) AND TS=(lung OR pulmonary OR bronchial)) AND TS=(cancer OR carcinoma OR neoplas* OR tumor* OR malignan* OR oncol*)) AND TS=(‘perioperative’ OR ‘neoadjuvant’ OR ‘adjuvant’)) AND TS=(pembrolizumab OR nivolumab OR atezolizumab OR durvalumab OR camrelizumab OR tislelizumab OR sintilimab OR ramucirumab OR oleclumab OR monalizumab OR danvatirsen OR relatlimab OR ipilimumab OR toripalimab OR ‘immune checkpoint inhibitors’ OR ‘immunotherapy agents’ OR ‘PD-1 inhibitor’ OR ‘PD-L1 inhibitor’ OR ‘immunotherapy’ OR ‘chemotherapy’ OR ‘Chemoradiotherapy’)) NOT TS=(retrospective study OR observational study) AND TS=(Human) NOT TS=(animal* OR mouse OR rat OR in vivo) NOT TS=(review OR news OR letter OR editorial OR case report OR systematic review OR meta-analysis) | |
| **Cochrane CENTRAL search query** | 773 |
| #1 MeSH descriptor: [Carcinoma, Non-Small-Cell Lung] explode all trees  #2 (lung OR pulmonary):ti,ab,kw  #3 (cancer OR carcinoma OR neoplas*):ti,ab,kw  #4 #2 AND #3  #5 #1 OR #4  #6 (perioperative OR neoadjuvant OR adjuvant):ti,ab,kw  #7 (pembrolizumab OR nivolumab OR atezolizumab OR durvalumab OR camrelizumab OR tislelizumab OR sintilimab OR toripalimab OR relatlimab OR ipilimumab OR 'anti-PDL1' OR 'anti-PD1' OR 'PD-1' OR PD-L1 OR 'Programmed Death 1' OR 'Programmed Cell Death 1 Receptor' OR 'Programmed Death-Ligand 1' OR 'immune checkpoint inhibitor' OR 'immune therapy' OR immunotherapy OR chemoimmunotherapy OR chemo-immunotherapy OR Chemoradiotherapy):ti,ab,kw  #8 #5 AND #6 AND #7  #9 (human OR humans):ti,ab,kw  #10 #8 AND #9 | |

**Table S2. Summary of Racial Distribution in Studies on Immunotherapy Checkpoint Inhibitors Combined with Chemotherapy**

| Registration Number | Author and Year | Race* | Treatment | Sample Size | MPR (%) | pCR (%) |
| --- | --- | --- | --- | --- | --- | --- |
| NCT03081689 | Provencio M, et al. 2019 | White | Nivolumab + Chemotherapy | 46 | 76 | 54.3 |
| NCT04606303 | Yan S, et al. 2023 | Asian | Toripalimab + Chemotherapy | 100 | 52 | 37 |
| NCT04304248 | Zhao Z, et al. 2021 | Asian | Toripalimab + Chemotherapy | 33 | 60.6 | 45.5 |
| ChiCTR2100044645 | Zhang Y, et al. 2022 | Asian | Camrelizumab + Chemotherapy | 26 | 38.4 | 19.2 |
| NCT04144608 | Zhang Y, et al. 2022 | Asian | Toripalimab + Chemotherapy | 33 | 45.5 | 33.3 |
| NCT04326153 | Sun C, et al. 2023 | Asian | Sintilimab + Chemotherapy | 30 | 33.3 | 16.6 |
| NCT02716038 | Shu C. A., et al. 2020 | White | Atezolizumab + Chemotherapy | 30 | 56.7 | 33.3 |
| NCT05024266 | Shan J, et al. 2024 | Asian | Tislelizumab + Chemotherapy | 35 | 68.6 | 40 |
| NCT06241807 | Cai G, et al. 2024 | Asian | Camrelizumab + Chemotherapy | 30 | 50 | 33.3 |
| NCT02998528 | Forde PM, et al. 2022 | Mixed | Nivolumab + Chemotherapy | 179 | 36.9 | 24 |
| NCT03425643 | Wakelee H, et al. 2023 | White | Pembrolizumab + Chemotherapy | 396 | 30.2 | 18.1 |
| NCT03800134 | Heymach JV, et al. 2023 | White | Durvalumab + Chemotherapy | 366 | 33.3 | 17.2 |
| NCT04025879 | Cascone T, et al. 2024 | White | Nivolumab + Chemotherapy | 229 | 35.4 | 25.3 |
| NCT04379635 | Yue D, et al. 2023 | Asian | Tislelizumab + Chemotherapy | 236 | 56.2 | 40.7 |
| NCT04158440 | Lu S, et al. 2024 | Asian | Toripalimab + Chemotherapy | 202 | 48.5 | 24.8 |
| NCT04338620 | J Lei, et al. 2023 | Asian | Camrelizumab + Chemotherapy | 43 | 65.1 | 32.6 |
| NCT03838159 | Provencio M, et al. 2023 | White | Nivolumab + Chemotherapy | 57 | 53 | 37 |

*Standardized categorization method: White Dominant (White ≥ 50%), Asian Dominant (Asian ≥ 50%), and Mixed (no single race ≥ 50%).
